# Plant Stress and Biotechnology

**DOI:** 10.1155/2013/170367

**Published:** 2013-04-30

**Authors:** Juan Francisco Jiménez Bremont, Margarita Rodríguez Kessler, Ji-Hong Liu, Sarvajeet S. Gill

**Affiliations:** ^1^Plant Biotechnology Lab, Division of Molecular Biology, IPICYT, 78216 San Luis Potosí, SLP, Mexico; ^2^Facultad de Ciencias, Universidad Autónoma de San Luis Potosí, 78290 San Luis Potosí, SLP, Mexico; ^3^National Key Laboratory of Crop Genetic Improvement, National Center of Crop Molecular Breeding, Huazhong Agricultural University, Wuhan 430070, China; ^4^Stress Physiology & Molecular Biology Lab, Centre for Biotechnology, MD University, Rohtak 124 001, Haryana, India

Nowadays plant biotechnology faces many important challenges, including the development of strategies to secure global food supply, the adequate acquisition and management of plant derived products for human subsistence (woods, pharmaceuticals, biofuels, etc.), as well as the development of plants that can cope with the constant effects of biotic and abiotic stress conditions in the era of changing climatic conditions. Being sessile, plants are able to adapt or acclimate after constant exposition to environmental stress conditions; nevertheless, in most cases growth rate and yield are reduced below optimum levels. Increases in global warming, climatic changes, soil degradation, and pollution, are affecting dramatically the plant kingdom. 

Advances in plant molecular biology and biotechnology have changed our capabilities for gene discovery, the study of tissue-specific promoters, the functional characterization of genes, gene pyramiding, and the generation of efficient methods for plant genetic transformation and/or plant genome manipulation. All of these beneficial applications offer the possibility to achieve pest resistance, abiotic stress tolerance, crop yield improvement, and excel food nutritional quality. 

In this regard, the understanding of plant molecular mechanisms underlying biotic and abiotic stress tolerance, the characterization of hormone mediated signaling pathways, and the molecular interaction and crosstalk among pathways will improve our knowledge in plant stress biology.

This special issue consists of six papers related to abiotic and biotic plant stress. Papers by M. Pérez-Clemente et al., L.-T. Yang et al., and A. S. Dubrovina et al. present interesting reviews of diverse topics such as plant response to stress, aluminium tolerance in higher plants, and pre-mRNA splicing under plant stress conditions, respectively. The following three are research papers focusing on identification of rice genes in response to heat stress (Y. Cao et al.), transcriptional profiling of canker-resistant transgenic sweet orange (X.-Z. Fu and J.–H. Liu), and characterization of the newly developed soybean cultivar (DT2008) under drought tolerance (C. V. Ha et al.). 

M. Pérez-Clemente et al. outline the main biotechnological approaches used to study plant stress responses, such as the “omics” technologies (genomics, proteomics, and metabolomics), and transgenic-based approaches. As well, considerable advances in plant physiology, genetics, and molecular biology are included, which have greatly improved our understanding of plant responses to abiotic stress conditions. 

A. S. Dubrovina et al. review recent data on alternative splicing in plant genes involved in stress signaling; in particular the authors focus on the occurrence, properties, and functional consequences of unconventional splicing and splicing-like events in plants. Precursor mRNAs with introns can undergo alternative splicing to produce multiple transcripts from the same gene by differential use of splice sites, thereby increasing the transcriptome and proteome complexity. In order to survive the stress conditions, plants actively employ pre-mRNA splicing as a mechanism to regulate expression of stress-responsive genes and reprogram intracellular regulatory networks. This review is attractive since it provides data on transcript diversity generated in response to environmental stresses, an aspect that could be important to plant biotechnology in terms of developing new strategies for crop breeding and protection.

L.-T. Yang et al. describe the main mechanisms related to aluminium tolerance in plants facing acidic soils, where aluminium toxicity is an important factor that limits crop productivity. Importance is given to the secretion of organic acid ions from plant roots and the possible mechanisms that regulate this process, including ion channels or transporters, internal concentration of organic acid ions, root plasma membrane H-ATPase, and temperature, among others. As well, transgenic plants attempting to increase the secretion and biosynthesis of organic acid anions as an approach for the acquisition of aluminium tolerance are discussed as potential research area.

In the research article presented by Y. Cao et al., the importance of studying heat stress responses in plant crops, such as rice, is emphasized, in particular, because of global warming and its increasing impact on crop production at the present time. In rice, several papers involving heat shock proteins (HSPs) and heat shock transcription factors in the heat stress response have been published; nevertheless, little is known about other genes induced under this condition. Based on cDNA-AFLP analysis, the authors identified 49 differentially expressed genes, including genes related to carbohydrate metabolism, photosynthesis and energy production, and amino acid and polyamine metabolism and transport, among others, as heat stress-responsive genes in rice. The possible implication of these genes in heat stress response is discussed.

In the research article presented by X.-Z. Fu and J.-H. Liu, they analyzed the global transcriptional profiling of transgenic sweet orange line (TG9) using the Affymetrix Citrus GeneChip microarray. TG9 line overexpresses *Malus domestica* spermidine synthase (MdSPDS1); it has increased levels of the polyamines, spermidine and spermine, and confers canker resistance. Nowadays, the gene regulation network in polyamine which involved plant pathogen response is largely unclear, particularly in perennial plants like citrus. A profile of genes up- and downregulated under high polyamine levels in the TG9 line is presented. It is hypothesized that genes implicated in stimulus response and immune system process, cell wall and transcriptional regulation, and cellular and metabolism processes, such as starch and sucrose metabolism, glutathione metabolism, biosynthesis of phenylpropanoids, and plant hormones play major roles in the canker resistance of TG9. 

In the research article presented by C. V. Ha et al., drought-tolerant phenotypes of DT2008 soybean variety and W82 soybean cultivar were compared by examining the dehydration-induced water loss and membrane stability of the shoot parts of young seedlings. DT2008 is an improved variety of soybean that shows enhanced drought tolerance and stable yield in field conditions. The authors propose that further comparisons between DT2008 and W82 cultivars using molecular approaches will enable the identification of mutations associated with drought tolerance in DT2008, which could be used to improve drought tolerance in soybean cultivars through genetic engineering. 

In the research article presented by C. V. Ha et al., they compared the drought-tolerant phenotypes of DT2008 soybean variety and W82 soybean cultivar by examining the dehydration-induced water loss and membrane stability of the shoot parts of the young seedlings. DT2008 is an improved variety of soybean that showed enhanced drought tolerance and stable yield in the field conditions. Molecular analysis between these soybean cultivars will enable the identification of mutations, which could be candidates for genetic engineering to improve drought tolerance in soybean cultivars.

